# Positioning of second‐line treatment for advanced gastric and gastroesophageal junction adenocarcinoma

**DOI:** 10.1002/cam4.941

**Published:** 2016-10-24

**Authors:** Carles Pericay, Fernando Rivera, Carlos Gomez‐Martin, Inmaculada Nuñez, Alejo Cassinello, Esteban Rodrigo Imedio

**Affiliations:** ^1^ Department of OncologySabadell University HospitalParc TauliSabadellSpain; ^2^ Department of Medical OncologyHospital Universitario SantanderSantanderSpain; ^3^ Gastrointestinal Cancer and Early Clinical and Translational Research Units12 de Octubre University HospitalMadridSpain; ^4^ Eli Lilly and CompanyAlcobendasMadridSpain

**Keywords:** Adenocarcinoma, gastric cancer, paclitaxel, ramucirumab, treatment

## Abstract

Tumors of the upper gastrointestinal tract are increasing in incidence; yet, approaches to the treatment of advanced gastric and/or gastroesophageal junction cancer vary widely, with no internationally agreed first‐line regimens. Recent clinical trials have shown that second‐line treatment is now possible for selected patients with advanced disease, and current data suggest that the combination of ramucirumab plus paclitaxel may become a standard of care in the second‐line setting for metastatic gastric cancer. Several prognostic factors have been identified for overall survival in the second‐line setting; this emphasizes the need for careful sequencing of all treatments to ensure that individual patients receive optimum care. This article reviews published data on the treatment of advanced gastric cancer, with a particular emphasis on second‐line chemotherapy, and suggests treatment sequences based on current understanding.

## Introduction

Gastric cancer is the fifth most common cancer in the world, with an estimated 951,000 new cases diagnosed in 2012 (6.8% of total cancer cases) 1, and the third leading cause of cancer death in both sexes worldwide, with 723,000 deaths (8.8% of total cancer deaths) estimated in that year. In Europe, 139,600 new cases were diagnosed and 107,300 patients died of gastric cancer in 2012 2. Gastric cancer is more frequent among males and its incidence increases with age, peaking between 65 and 74 years of age 1.

Geographically, almost three quarters of all gastric cancer cases occur in developing countries; it has particularly high incidence rates in Eastern Asia and South America 1. Incidence rates are comparatively low in many developed regions such as North America, Western Europe, and Australia/New Zealand, thought to be due to declining chronic *Helicobacter pylori* infection incidence in the Western world 3. Nevertheless, in these areas, the incidence of tumors located in the gastric cardia and gastroesophageal junction has increased in past decades and is linked to risk factors such as obesity and gastroesophageal reflux disease 4.

Surgical resection is currently the only curative treatment option for gastric cancer; however, ~50% of patients have metastatic disease at the time of diagnosis and chemotherapy is the mainstay of palliation in this setting 5. The estimated 5‐year survival rate in the USA is 29%; for those with stage IV disease, it is 4% 6.

Best supportive care (BSC) plus chemotherapy has been shown to be more effective than BSC alone in patients with advanced gastric cancer, with combination chemotherapy more effective than single‐agent treatment 7. Now, an increasing number of patients are candidates for second‐line treatment 8, and phase III trials 9, including two involving a total of 1020 participants 10, 11, and meta‐analyses 12, 13 have shown the potential benefit of second‐line treatment options. Despite these improvements, there are still no standardized treatment approaches for those with advanced disease, and optimal management is under debate 3.

This article reviews the published data on the treatment of advanced unresectable gastric cancer, with a particular emphasis on second‐line chemotherapy, and suggests treatment sequences based on current disease understanding.

## First‐Line Chemotherapy

### Current treatment options in first line

Patients with unresectable or metastatic gastric and/or gastroesophageal junction adenocarcinoma are candidates for chemotherapy‐based palliative treatment only; choice of chemotherapy at this stage is largely based on performance status (PS), organ function 14, and physician preference 3. First‐line treatments include platinums and fluoropyrimidines, either alone or in combination, sometimes with the addition of a third drug such as epirubicin or a taxane 3. Combination treatment is more worthwhile than monotherapy with any agent; results from pivotal phase III randomized controlled trials (RCTs) investigating different combination regimens are summarized in Table 1. A doublet of a platinum compound and a fluoropyrimidine is regarded as an acceptable standard first‐line option 15. A recent Cochrane review 16 showed a benefit for chemotherapy versus BSC on overall survival (OS) (hazard ratio [HR]: 0.37, 95% confidence interval [CI]: 0.24–0.55, 184 participants) and a survival benefit for combination chemotherapy compared with single‐agent 5‐fluorouracil (5‐FU) (HR: 0.82, 95% CI: 0.74–0.90, 1914 participants). More recently, triple‐agent regimens resulted in small improvements in OS versus doublet therapy, but their use is not globally accepted 15 and is associated with an increase in serious side effects 15.

**Table 1:** Major phase III trials for first‐line treatment in advanced gastric and/or esophageal junction cancer

Reference	Agents	Patients (randomized) (*N*)	Median OS (months)/comments
Doublet regimens			
Al‐Batran 2008 53	FLO vs. FLP	220	10.7 vs. 8.8; *P* = NS.In patients >65 years 13.9 vs. 7.2; *P* *=* 0.081
Kang 2009–ML 17031 54	CX vs. CF	316	10.5 vs. 9.3 (unadjusted HR: 0.85, 95% CI: 0.64–1.13, *P* = 0.008 [for noninferiority])
Ajani 2010 FLAGS (Western study) 55	S‐1 + cisplatin vs. infusional fluorouracil + cisplatin	1053	8.6 vs. 7.9 (HR: 0.92, 95% CI: 0.80–1.05; *P* = 0.20).Significant safety advantages with cisplatin/S‐1
Koizumi 2008 SPIRITS (Asian study) 56	S‐1/cisplatin vs. S‐1 alone	298	13.0 vs. 11.0 months (HR: 0.77, 95% CI: 0.61–0.98; *P* = 0.04)
Triplet combinations			
Webb 1997 26	ECF vs. FAMTX	274	8.9 vs. 5.7; *P* = 0.0009
Van Cutsem 2006–V325 30	DCF vs. CF	447	9.2 vs. 8.6; *P* = 0.02Toxicity worse with DCF
Cunningham 2008–REAL‐2 29	ECF or ECX or EOF or EOX	1002	9.9, 9.9, 9.3, and 11.2 in ECF, ECX, EOF, and EOX groups, EOX vs. ECF 11.2 vs. 9.9 (HR: 0.80, 95% CI: 0.66–0.97; *P* = 0.02)
Targeted treatments			
Bang 2010–ToGA 18	Capecitabine/cisplatin or fluorouracil/cisplatin ± trastuzumab	594	11.1 vs. 13.8 chemotherapy alone vs. chemotherapy + trastuzumab (HR: 0.74, 95% CI: 0.60–0.91; *P* = 0.0046)
Ohtsu 2011–AVAGAST 20	Capecitabine/cisplatin ± bevacizumab	774	10.1 vs. 12.1 chemotherapy alone vs. chemotherapy + bevacizumab (HR: 0.87, 95% CI: 0.73–1.03; *P* = 0.1002)
Waddell 2013–REAL‐3 31	EOX ± panitumumab	553	11.3 vs. 8.8 chemotherapy alone vs. chemotherapy + panitumumab (HR for OS 1.37; *P* = 0.013)
Lordick 2013–EXPAND 21	Capecitabine/cisplatin ± cetuximab	904	PFS 5.6 vs. 4.4 chemotherapy alone vs. chemotherapy + cetuximab (HR: 1.09, 95% CI: 0.92–1.29; *P* = 0.32)

CF, cisplatin, fluorouracil; CI, confidence interval; CX, cisplatin, capecitabine; DCF, docetaxel, cisplatin, fluorouracil; ECF, epirubicin, cisplatin, fluorouracil; ECX, epirubicin, cisplatin, capecitabine; EOF, epirubicin, oxaliplatin, fluorouracil; EOX, epirubicin, oxaliplatin, capecitabine; FAMTX, fluorouracil, doxorubicin, methotrexate; FLO, fluorouracil, leucovorin, oxaliplatin; FLP, fluorouracil, leucovorin, cisplatin; HR, hazard ratio; NS, not significant; OS, overall survival; PFS, progression‐free survival.

The use of targeted treatments in the first‐line therapy of advanced gastric cancer is also evolving and paving the way for personalized medicine 17, although human epidermal receptor type 2 (HER2) status is currently the only validated molecular marker to influence decision‐making in advanced disease 3. Trastuzumab, in combination with capecitabine and cisplatin or 5‐FU and cisplatin, significantly improved survival in patients with overexpression of HER2 18 (Table 1), but only 20% of gastric cancers and 30% of gastroesophageal cancers overexpress HER2 19. Neither bevacizumab nor the antiendothelial growth factor receptor antibody cetuximab has showed any survival advantage when added to a fluoropyrimidine and cisplatin regimen versus this same regimen alone 20, 21 (Table 1).

### Differences in treatment approach

There is no internationally agreed standard first‐line regimen for advanced disease and treatment approaches differ internationally 22. In Europe, epirubicin, cisplatin, 5‐FU (ECF) is the reference regimen, whereas in the USA, cisplatin–fluoropyrimidine combinations and docetaxel, cisplatin, 5‐FU (DCF) triplets are most often used. In Japan, and the rest of Asia, cisplatin plus S‐1 has recently become the standard.

Regional differences are evident between Asian and Western countries in epidemiology, treatment regimens, and outcomes (both efficacy and safety) 17. Some differences are due to well‐documented ethnicity‐related variations in drug metabolism 23. The metabolism of S‐1 displays ethnic differences, leading to differential dose tolerances and toxicity; in Western patients, the tolerable dose is substantially lower than that in Asian populations 24. In addition, the incidences of grade 3–4 neutropenia and grade 3–4 diarrhea in association with first‐line chemotherapy are 8.2% and 2.1% lower in trials in Asia compared with trials in non‐Asian countries 25.

### Role of triplet therapy

Evidence for the efficacy of an anthracycline‐based triplet regimen was obtained from a phase III study comparing ECF with fluorouracil, doxorubicin, and methotrexate (FAMTX) in advanced esophagogastric cancer 26. Overall response rate was higher with ECF (45%, 95% CI: 36–54 vs. 21%, 95% CI: 13–29; *P* = 0.0002). ECF was also superior in terms of survival and global quality of life (QoL). These results were strengthened by a prospectively randomized study that showed ECF to have equivalent efficacy to mitomycin C, cisplatin, and 5‐FU (MCF) but with superior QoL 27. A meta‐analysis described a significant survival advantage of combining 5‐FU/cisplatin (CF) regimens with anthracyclines versus those without anthracyclines (HR: 0.77, 95% CI: 0.62–0.95) 7.

The epirubicin, oxaliplatin, capecitabine (EOX) regimen is another option to be considered 28, based on results from the REAL‐2 study 29. Capecitabine was noninferior to 5‐FU (HR: 0.86, 95% CI: 0.8–0.99) and oxaliplatin was noninferior to cisplatin (HR: 0.92, 95% CI: 0.8–1.1). Median survival was higher for EOX (11.2 months) than for ECF (9.9 months) (*P* = 0.02). The toxic effects of capecitabine and 5‐FU were similar. Oxaliplatin caused less neutropenia, alopecia, renal toxicity, and thromboembolism, but more diarrhea and neuropathy than cisplatin.

In the USA, the DCF regimen is favored for patients who can tolerate it. In the V325 trial 30, DCF was superior to CF for time to progression (TTP) [5.6 vs. 3.7 months; HR: 1.473, 95% CI: 1.189–1.825; *P* = 0.004], median OS (9.2 vs. 8.6 months; *P* = 0.02), and response rates (37% vs. 25%; *P* = 0.01). However, grade 3–4 neutropenia (82% vs. 57%) and all grades of febrile neutropenia (29% vs. 2%) were significantly higher with DCF 30. Modified DCF regimens continue to be explored in an attempt to maintain efficacy while reducing excessive toxicity 31. For example, the so‐called miniDOX regimen, which involves reduced doses of docetaxel, oxaliplatin, and capecitabine, has shown promising results in patients considered not suitable for treatment with the standard DCF regimen due to poor PS, weight loss, and/or age 32. In addition, the ML21085‐ACROSS trial using modified docetaxel, cisplatin, capecitabine (DCX) in patients with Eastern Cooperative Oncology Group (ECOG) PS 0–1 demonstrated a survival benefit (OS 11.86 months), with low grade 3 toxicity (mucositis 11.4%, febrile neutropenia 3.0%) 33. A third option is the so‐called FLOT (oxaliplatin, docetaxel, leucovorin, fluorouracil) regimen. Objective response rates of 55% and 58%, and median OS durations of 10 and 11 months have been reported in two studies; the most common grade 3/4 adverse events were neutropenia (45% and 48%), leukopenia (26% and 28%), and diarrhea (15% in both studies) 34, 35.

Three studies have explored irinotecan as first‐line treatment, including one meta‐analysis. The findings of two RCTs suggest there is no survival benefit associated with irinotecan, although it may offer a platinum‐free treatment alternative. In the first study 36, irinotecan combined with 5‐FU and folinic acid (IF) was associated with a nonsignificant improvement in TTP and OS compared with CF. In the second study, ECX followed by the FOLFIRI regimen (irinotecan, 5‐FU, folinic acid) was compared with the reverse sequence. FOLFIRI had no impact on OS, disease‐free survival, or response rate 37. However, a meta‐analysis of 10 RCTs concluded that there was strong evidence for a survival benefit with irinotecan‐containing regimens as first‐line treatment in patients with advanced gastric cancer. A clear advantage of irinotecan‐containing over non‐irinotecan‐containing regimens was not established 38.

In the current National Comprehensive Cancer Network guidance for advanced disease 39, two‐drug regimens are preferred as first‐line therapy because of lower toxicity; three‐drug regimens are reserved for medically fit patients with good ECOG PS and access to frequent toxicity evaluations. The preferred regimens are DCF, ECF, or modifications of these.

**Figure 1:**
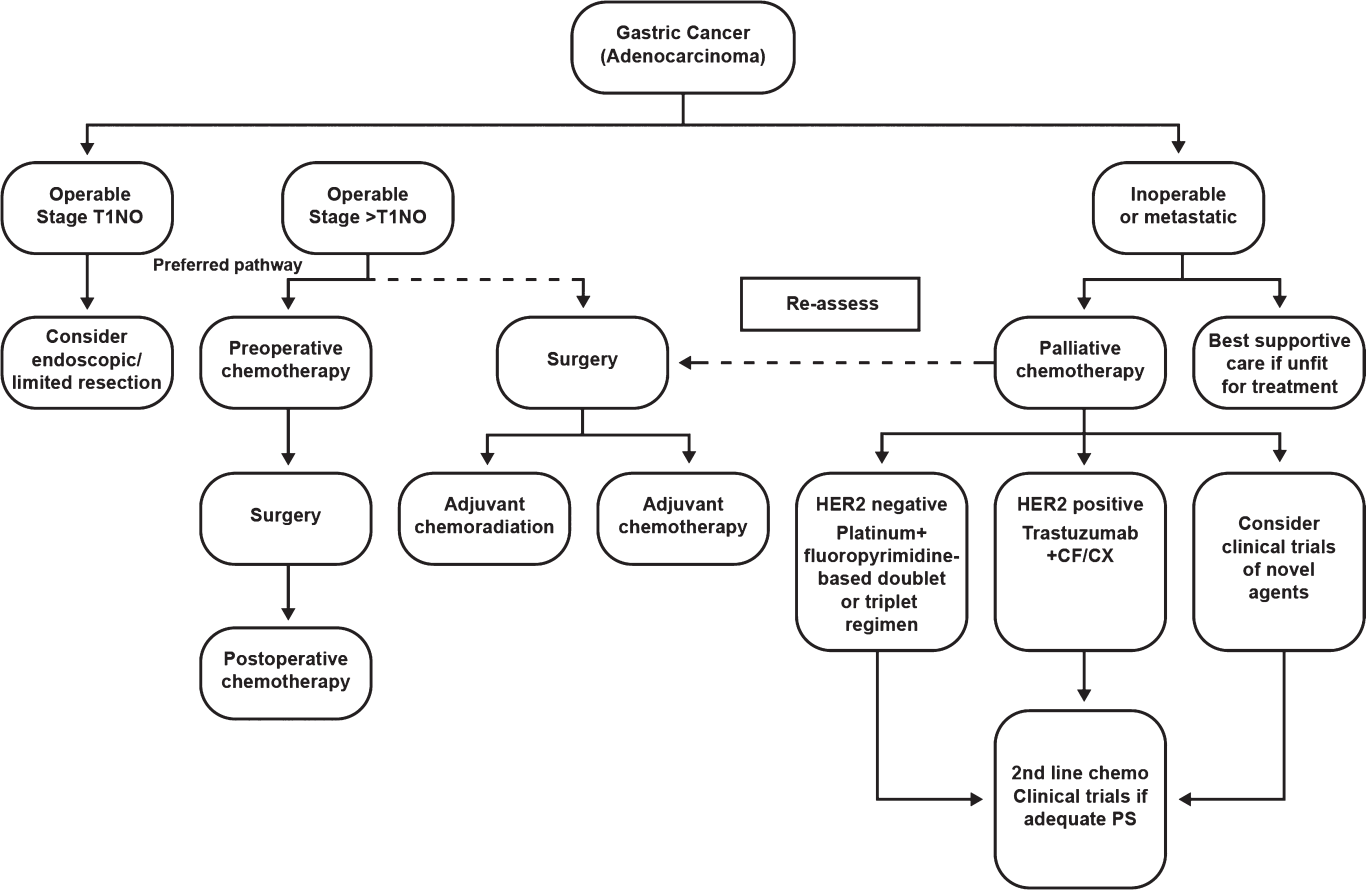
ESMO‐ESSO‐ESTRO guidance for the treatment of gastric cancer. Reproduced from Waddell et al. 31 with permission from Oxford University Press. CF, 5‐fluorouracil/cisplatin; CX, cisplatin, capecitabine; ESMO, European Society for Medical Oncology; ESSO, European Society of Surgical Oncology; ESTRO, European Society of Radiotherapy and Oncology; HER, human epidermal receptor; PS, performance status.

Figure 1 outlines current guidance from the European Society for Medical Oncology/European Society of Surgical Oncology/European Society of Radiotherapy and Oncology (ESMO‐ESSO‐ESTRO). For HER2‐negative patients, a platinum/fluoropyrimidine‐based doublet or triplet regimen is recommended; for HER2‐positive patients, trastuzumab plus CF/CX is recommended 31.

## Second‐Line Chemotherapy

The lack of universally accepted standard therapies beyond first line may have contributed to the poor survival rates in advanced gastric cancer seen until relatively recently. However, increasing evidence now suggests that second‐line therapies may improve OS 40, and recent results from two large phase III studies with the monoclonal antibody ramucirumab are particularly robust 10, 11.

Three phase III studies of irinotecan or docetaxel support the use of second‐line chemotherapy in patients who are fit enough. The first was a South Korean study that compared either irinotecan or docetaxel with BSC: 41 results showed a median OS of 5.1 versus 3.8 months (*P* = 0.004) for chemotherapy versus BSC. The second was a German study that compared irinotecan with BSC 9. This study was closed prematurely due to poor accrual. Median OS was 4.0 months versus 2.4 months in the irinotecan and placebo arms, respectively (*P* = 0.012). The third was a UK multicenter study (COUGAR‐02) that examined the effects of adding docetaxel to BSC as second‐line treatment 42. Median OS in the docetaxel group was 5.2 months versus 3.6 months in the BSC arm (*P* = 0.01). A meta‐analysis of data from these three studies showed a statistically significant improvement in OS with second‐line chemotherapy in advanced gastric cancer (*P* < 0.0001) 12.

In another trial comparing paclitaxel with irinotecan in Japanese patients, both drugs had similar positive effects on survival 43, with a median OS of 9.5 versus 8.4 months, respectively (*P* *=* 0.38).

More recently, two large international phase III multicenter studies (REGARD and RAINBOW) have investigated the potential for second‐line treatment with ramucirumab, a fully human monoclonal antibody against the vascular endothelial growth factor receptor (VEGFR)‐2. The primary endpoint was OS in both trials. REGARD compared monotherapy with ramucirumab and BSC versus placebo and BSC in patients with advanced gastric or gastroesophageal junction adenocarcinoma 10. Eligible patients had disease progression after first‐line platinum‐containing or fluoropyrimidine‐containing chemotherapy for metastatic disease, ECOG PS 0–1, and measurable disease (defined by Response Evaluation Criteria In Solid Tumors [RECIST] version 1.0) 44 or evaluable disease. Patients were randomized 2:1 to receive BSC plus either intravenous ramucirumab 8 mg/kg or placebo once every 2 weeks. A total of 355 patients were included (ramucirumab *n* = 238; placebo *n* = 117).

**Figure 2:**
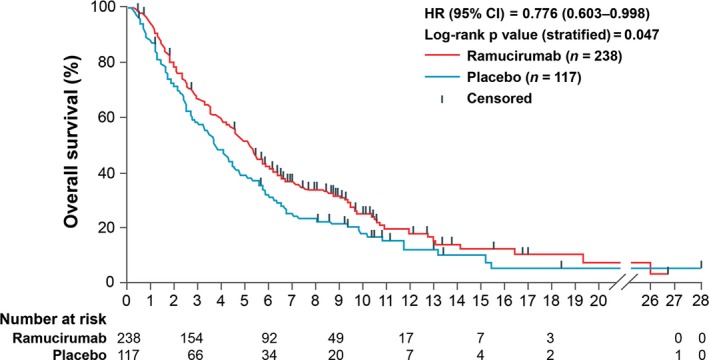
Kaplan–Meier estimates of overall survival. Reprinted from The Lancet 10 with permission from Elsevier. Survival in patients receiving ramucirumab monotherapy for previously treated advanced gastric or gastroesophageal junction adenocarcinoma versus placebo from the international, randomized, multicenter, placebo‐controlled, phase III REGARD trial. CI, confidence interval; HR, hazard ratio.

Median OS was 5.2 months (interquartile range [IQR]: 2.3–9.9) in the ramucirumab group and 3.8 months (1.7–7.1) in the placebo group (HR: 0.776, 95% CI: 0.603–0.998; *P* = 0.047) (Fig. 2). Three significant independent predictors for reduced OS were ECOG PS ≥1, esophagogastric junction location of the primary tumor, and presence of peritoneal metastases. Treatment with ramucirumab reduced the risk of disease progression or death from any cause by 52%. Median progression‐free survival (PFS) was 2.1 months (IQR: 1.3–4.2) in patients receiving ramucirumab and 1.3 months (IQR: 1.1–2.1) in those receiving placebo.

Ramucirumab appeared to be well tolerated. Rates of serious adverse events were similar between ramucirumab and placebo recipients: 57% versus 58%, respectively, experienced grade ≥3 adverse events. Grade 4 hypertension was not observed. Median time to deterioration in ECOG PS to ≤2 was 5.1 months in the ramucirumab group and 2.4 months in the placebo group 10, 45.

The RAINBOW trial investigated ramucirumab in combination with paclitaxel versus placebo plus paclitaxel as second‐line treatment 11. Eligible patients had advanced gastric or gastroesophageal junction adenocarcinoma and disease progression following platinum plus fluoropyrimidine with or without an anthracycline. Eligibility criteria were similar to those of REGARD. Patients were randomized 1:1 to receive either intravenous ramucirumab 8 mg/kg (*n* = 330) or placebo (*n* = 335) on days 1 and 15, plus intravenous paclitaxel 80 mg/m^2^ on days 1, 8, and 15 of a 28‐day cycle.

Disease progression was experienced by 69% of patients while still receiving first‐line therapy, and many had other poor prognostic factors, including poorly differentiated tumors, disease progression within 6 months after the start of the previous therapy, at least three metastatic sites, presence of primary tumor, peritoneal metastases, or presence of ascites.

**Figure 3:**
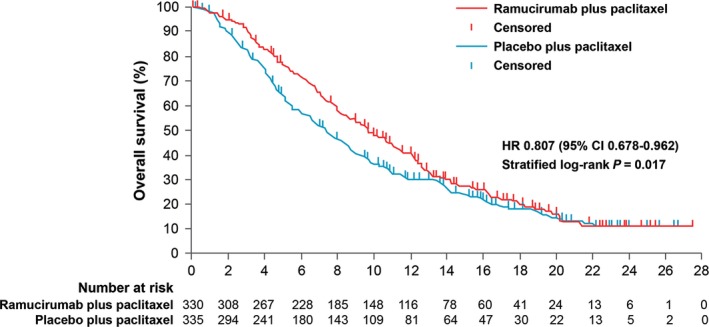
Kaplan–Meier estimates of overall survival. Reprinted from The Lancet 11 with permission from Elsevier. Survival in patients receiving ramucirumab plus paclitaxel for previously treated advanced gastric or gastroesophageal junction adenocarcinoma versus placebo plus paclitaxel, from the double‐blind, randomized phase III RAINBOW trial. CI, confidence interval; HR, hazard ratio.

OS was significantly longer in ramucirumab versus placebo recipients (median 9.6 months, 95% CI: 8.5–10.8, vs. 7.4 months, 95% CI: 6.3–8.4; HR: 0.807, 95% CI: 0.678–0.962; *P* = 0.017) (Fig. 3). Stepwise Cox proportional modeling with inclusion of all prespecified factors identified seven significant independent predictors for improved survival. After adjustment, the HR for OS with ramucirumab plus paclitaxel versus placebo plus paclitaxel was 0.745 (95% CI: 0.626–0.888; *P* = 0.0010). ECOG PS, geographic region, and presence of ascites were the strongest predictors for survival. Baseline and end‐of‐treatment results were similar between treatment groups for global QoL from the European Organisation for Research and Treatment of Cancer (EORTC) QoL questionnaire (QLQ‐C30) and index scores from the EuroQOL five‐dimension questionnaire (EQ‐5D‐3L), indicating that QoL was maintained on treatment with ramucirumab plus paclitaxel.

Grade ≥3 adverse events in >5% of patients in the ramucirumab plus paclitaxel group included neutropenia (41% vs. 19%, respectively), leucopenia (17% vs. 7%), hypertension (14% vs. 2%), fatigue (12% vs. 5%), anemia (9% vs. 10%), and abdominal pain (6% vs. 3%). The incidence of grade ≥3 febrile neutropenia was similarly low in both groups (3% vs. 2%).

RAINBOW is the largest trial in second‐line gastric cancer and the first study to report a survival benefit with a VEGFR‐2‐targeted antibody in combination with chemotherapy in advanced gastric cancer. Taken together, the results of REGARD and RAINBOW show that ramucirumab can significantly prolong survival and suggest that ramucirumab offers an important new treatment option in this patient population 3.

## Treatment Sequencing

### Prognostic patient factors

Evidence of benefit with second‐line treatment highlights the need for careful treatment decision‐making in the first‐line setting based on individual patient factors. Various factors have been found to influence the survival of patients undergoing second‐line therapy, and these can help guide and inform decisions on optimal treatment sequences. Factors include ECOG PS, potential cumulative toxicity from previous treatments (especially in those patients with low PS), extent of disease, lack of cross‐resistance with drugs previously used, the evidence available from specific treatment sequencing, and the response to first‐line treatments.

In RAINBOW 10, the strongest independent predictors of survival were ECOG PS, geographic region, and presence of ascites. Wilke and colleagues 11 speculated that geographic regional differences in OS might be due to the much higher use of poststudy discontinuation treatment in Asia (about 70%) compared with other regions (about 40%). A pooled analysis of REGARD and RAINBOW examined 41 key baseline covariates 46 and identified 12 independent factors associated with improved OS (five clinical; seven laboratory), as outlined in Table 2.

**Table 2:** Poor prognostic factors for overall survival in the second‐line setting 46

Poor prognostic factors	Hazard ratio (99% CI) for mortality
Peritoneal metastasis	1.49 (1.22–1.83)
Time‐to‐progressive disease on prior therapy <6 months	1.35 (1.10–1.66)
Eastern Cooperative Oncology Group performance status ≥1	1.39 (1.12–1.73)
Tumor differentiation (poor/unknown)	1.33 (1.08–1.64)
Primary tumor present	1.31 (1.05–1.62)
Alkaline phosphatase (high)	1.28 (1.03–1.60)
Sodium (low)	2.04 (1.54–2.71)
Lactate dehydrogenase (high)	1.31 (1.05–1.63)
Aspartate aminotransferase (high)	1.37 (1.06–1.76)
Albumin (low)	1.33 (1.07–1.65)
Lymphocytes (low)	1.31 (1.05–1.63)
Neutrophils (high)	1.52 (1.17–1.99)

Other studies have also identified potentially important prognostic factors. An Italian study demonstrated five statistically significant factors associated with poor survival outcomes in second‐line treatment:^47^ ECOG PS 2, hemoglobin ≤11.5 g/L, carcinoembryonic antigen >50 ng/mL, three or more metastatic sites of disease, and TTP under first‐line chemotherapy of ≤6 months. In a retrospective study from Japan, variables independently associated with shorter survival were ECOG PS 2, serum albumin level <3.5 g/dl at initiation of second‐line chemotherapy, and TTP of <170 days under first‐line chemotherapy 48. In a similar retrospective study, both mild and severe neutropenia after second‐line treatment with paclitaxel were associated with reduced risk of death; HR for death was 0.61 (95% CI: 0.41–0.88; *P* = 0.009) for patients with severe neutropenia 49.

Kanagavel et al. 50 developed a prognostic model in patients treated with second‐line chemotherapy based on the identification of three independent prognostic factors from multivariate analysis: ECOG PS 0–1 (HR: 2.3, 95% CI: 1.7–5.4), hemoglobin level ≥10 g/dl (HR: 2.2, 95% CI: 2.1–2.4), and TTP under first‐line therapy ≥5 months (HR: 0.5, 95% CI: 0.3–0.8). They divided patients into good‐, intermediate‐, and poor‐risk groups and found median survival to be 13.5, 6.0, and 2.9 months, respectively (*P* = 0.00001).

A recent meta‐analysis 51 aimed to estimate the potential survival of patients with advanced gastric cancer undergoing second‐line treatment after failure with first‐line treatment and to analyze the differential role of chemotherapy versus targeted agents. Results showed that any therapy was more effective than BSC and that, when populations were divided based on type of treatment, chemotherapy decreased the risk of death by 27% (HR: 0.73, 95% CI: 0.58–0.96), ramucirumab decreased the risk by 22% (HR: 0.78, 95% CI: 0.60–1.00), and everolimus had no significant effect on OS (HR: 0.90, 95% CI: 0.75–1.08). For patients with ECOG PS ≥1, ramucirumab offered significant benefit, with a reduction in the risk of death of 32% (HR: 0.68, 95% CI: 0.51–0.92; *P* = 0.04), showing that second‐line ramucirumab could be useful for patients with suboptimal PS. In patients with ECOG PS 0, both chemotherapy and ramucirumab significantly reduced the risk of death versus BSC. In addition, in RAINBOW, ramucirumab plus paclitaxel showed a survival benefit versus paclitaxel and placebo (median OS 9.6 vs. 7.4 months) 11.

### Selecting treatment sequence

Overall, the evidence suggests that ramucirumab plus paclitaxel could be regarded as a new standard second‐line treatment for advanced gastric cancer, as this combination is currently the most active treatment in this setting 11. Carefully planned treatment sequencing will be increasingly relevant to maximize success in a continuum of care. As previously discussed, second‐line chemotherapy can offer the best results in patients with the fewest negative prognostic factors; hence, choosing first‐line treatments with the lowest potential for toxicity and those that preserve ECOG PS might be advisable.

Currently, there are several different regimens for first‐line treatment: (1) doublets with a platinum compound (cisplatin or oxaliplatin) and a fluoropyrimidine (5‐FU, capecitabine, or S‐1); (2) triplets adding epirubicin or docetaxel; or (3) doublets with irinotecan and a fluoropyrimidine. Before choosing a first‐line regimen, we should consider the following:

1. Toxicity – choice of a more toxic regimen (e.g., triplets, mainly with docetaxel) increases cumulative toxicities from aggressive first‐line therapy and could reduce the option of using a second‐line treatment. Control of toxicity is mandatory for patients treated with a triplet regimen.
2. Use of taxanes – use of docetaxel in first‐line treatment could increase resistance to second‐line therapy; no data are available for second‐line ramucirumab–paclitaxel therapy in this setting.
3. Use of irinotecan‐based regimens – there are no data regarding second‐line ramucirumab–paclitaxel therapy in patients treated with a first‐line irinotecan‐based regimen. Also, irinotecan could be a feasible option for second‐line therapy in patients with intermediate ECOG PS or for third line (after ramucirumab–paclitaxel) in patients with a good PS.

To the best of our knowledge, only two published trials have specifically compared chemotherapy sequencing in gastric cancer patients 37, 52. Neither study found differences in OS between sequences.

Based on these considerations, a platinum–fluoropyrimidine doublet (or a triplet with epirubicin) therapy followed by second‐line paclitaxel–ramucirumab (in patients with a good ECOG PS) or by second‐line monotherapy with ramucirumab, irinotecan, or a taxane (in patients with intermediate PS) could be the best sequence of treatment for patients with advanced gastric cancer. In patients who maintain PS after second‐line treatment, even a third‐line therapy (with irinotecan, apatinib, or a taxane if not used previously) could be considered. Further data could be provided in future by studies such as the ongoing TO‐TAS‐102‐302 (ClinicalTrials.gov Identifier: NCT02500043), which is exploring TAS‐102 (a combination of the oral nucleoside analog trifluridine plus tipiracil, a thymidine phosphorylase inhibitor) in patients with metastatic gastric cancer who have previously received at least two prior regimens for advanced disease.

It is important to remark that, to be able to offer patients a second‐line therapy, toxicities and response (and/or progression) should be properly evaluated during first‐line treatment. Inadequate management of cumulative toxicities during first‐line therapy will impair the likelihood of using an active treatment in second line. If response is not evaluated in a timely manner, disease progression during first line may go unnoticed, and the patient may suffer deterioration precluding the use of any further treatment.

## Conclusion

Second‐line treatment with ramucirumab plus paclitaxel is likely to be regarded as a new standard for patients with advanced gastric and/or esophageal junction cancer, with good ECOG PS who have progressed after first‐line chemotherapy. Oncologists now need to choose first‐line regimens that combine good activity with good tolerability and fewer toxic effects for these patients to optimize the potential benefits of second‐line treatment. In this setting, a platinum–fluoropyrimidine doublet (or a triplet with epirubicin) could be the best first‐line sequence. Further studies are now needed to investigate the most favorable treatment sequences for advanced gastric cancer, and physicians should be alert to the eligibility of patients for enrollment in appropriate clinical trials.

## Conflict of Interest

FR has received personal fees from Amgen, Bayer, Roche and Sanofi for advisory boards and research funds, and has received personal fees from Eli Lilly and Company for advisory boards. CG‐M has received fees from Lilly Spain S.A. ERI, IN, and AC are full‐time employees of Eli Lilly and Company. CP has no conflicts of interest to declare.
